# Knowledge, attitudes, and behaviour of college students in disposing used masks during the COVID-19 pandemic in DKI Jakarta Province

**DOI:** 10.12688/f1000research.130691.1

**Published:** 2023-05-17

**Authors:** Avicenna Inovasanti, Dewi Susanna, Sandeep Poddar, Ema Hermawati, Aria Kusuma

**Affiliations:** 1 Department of Environmental Health, Faculty of Public Health, Universitas Indonesia, Depok, West Java, 16424, Indonesia; 2 Research and Innovation, Lincoln University College, Petaling Jaya, Selangor, 47301, Malaysia; 3 Health Development Policy Agency, Ministry of Health of Indonesia, Jakarta Selatan, Jakarta, 12950, Indonesia

**Keywords:** COVID-19, knowledge, attitudes, behaviour, sociodemographic, college students, mask waste management, living alone

## Abstract

Background: The COVID-19 pandemic has increased the need for and production of masks. This creates a problem, namely the generation of mask waste which ends up in the environment without being managed. This research was conducted to determine the relationship between knowledge, attitudes, and sociodemographic factors with college student behaviours in managing household mask waste in Daerah Khusus Ibukota (DKI) Jakarta Province. Methods: This study used a quantitative approach and cross-sectional study design. Data collection was carried out using an online questionnaire consisting of the respondents’ sociodemographic, knowledge, attitudes, and behaviours. Results: The majority of students had high knowledge (63.3%), positive attitudes (52.5%), and good behaviours (50.6%). Statistically, there was a significant relationship between knowledge and behaviours (p-value = 0.022), but there was no significant relationship between attitudes and behaviours (p-value = 0.269). In addition, the sociodemographic factor variables showed a significant relationship between place of residence and behaviours (p-value = 0.008). However, there was no significant relationship between age, gender, education, and study program groups and behaviours (p value> 0.05). Multivariate analysis showed that living with family was a dominant factor for bad behaviours (OR 1.664, 95% CI=1.124-2.464), and the second risk factor was the low level of knowledge has a significant relationship with the behaviours of mask waste management at home (OR=1.559, 95% CI=1.044-2.330). Conclusions: Students who live alone also show better behaviour compared to students who live with their families. The place of residence variable has the greatest influence on the behaviour of mask waste management at the household, followed by the knowledge variable.

## Introduction

The world still tries to face a major disaster consequence of the COVID-19 pandemic. Preventive measures continue to be taken to control high transmission and spread, one of the effective steps that can be taken is to use a mask ( Chowdhury, Chowdhury and Sait, 2020) ( World Health Organization, 2020a). The decreasing cases of COVID-19 have made the Indonesian government decide to revoke *Pemberlakuan Pembatasan Kegiatan Masyarakat* (PPKM) under the Instructions of the Minister of Home Affairs Numbers 50 and 51 of 2022, but the government still advises the public to continue using masks properly ( Menteri dalam Negeri Republik Indonesia, 2022a, 2022b). The high demand for masks has encouraged mass production and has experienced extraordinary growth ( Du, Huang and Wang, 2022).

The generation of plastic waste in the world continues to increase due to masks amid the COVID-19 pandemic. If not handled properly, this mask waste may still carry pathogenic contaminants that can potentially become hazardous waste ( Du, Huang and Wang, 2022). Disposable masks that are made from plastic base materials end up in public landfills and oceans without being managed, causing a big issue of the medical waste pollution in the environment ( Saadat, Rawtani and Mustansar, 2020). According to Ocean Asia 2020 report, it is estimated that as much as 1.6 billion face masks globally end up in the oceans ( Bondaroff and Cooke, 2020). Ministry of Environment and Forestry data shows that the mask waste generated in Indonesia has reached more than 3 million tons in 2020 ( Meidiana, 2021). DKI Jakarta Province in 2020 is estimated to be the highest producer of medical waste of 1,125,154 kg per day. Research also estimates that, on average, people in DKI Jakarta Province produce 52.88 to 79.32 tons of mask waste per day ( Sari *et al.*, 2021).

In dealing with this issue, it is necessary to make prevention efforts by involving the community. The Indonesian government has made policies and guidelines for managing household mask waste ( Amalia *et al.*, 2020; Hesti, 2020). Disposable mask waste management is regulated starting from collecting and separating mask waste from domestic waste, spraying disinfectant on masks, changing the shape of masks before being disposed of by tearing or cutting them, wrapping mask waste in plastic bags or throwing it into a closed trash can, and washing hands with soap and running water or use hand sanitizer after disposing of mask waste ( Kementerian Kesehatan RI, 2020; Menteri Lingkungan Hidup dan Kehutanan, 2020; Menteri Lingkungan Hidup dan Kehutanan Republik Indonesia, 2021).

Obedience in using masks is essential and heavily influenced by people’s knowledge, attitudes, and behaviours. According to Sukesih *et al.* (2020), a high level of knowledge encourages positive attitudes and good behaviours ( Sukesih *et al.*, 2020). By analysing the level of knowledge, attitudes, and behaviours, students can become important players in planning and ensuring preventive actions for the younger generation in preventing COVID-19 ( Olaimat *et al.*, 2020; Saefi *et al.*, 2020). Other factors that influence public health behaviours are sociodemographic factors such as differences in age, gender, education, occupation, and region of origin ( Moudy and Syakurah, 2020).

Several studies have been conducted on the handling of COVID-19. However, the indirect impact of the pandemic still tends to be ignored such as managing household mask waste. College students become the object of this research as a generation with a high level of education and independency. Therefore, this study aimed to analyse the relationship between knowledge, attitudes, and sociodemographic factors with student behaviours in managing household mask waste in DKI Jakarta Province.

## Methods

### Research design

This research was a quantitative study with a cross-sectional approach because the measurements of the dependent and independent variables were carried out only once at the same time. This study aimed to analyse the relationship between knowledge, attitudes, and sociodemographic factors with student behaviours in managing household mask waste in DKI Jakarta Province. The dependent variable studied was student behaviours in managing household mask waste and the independent variables were sociodemographic characteristics (age, gender, education, science groups, and place of residence), students’ knowledge, and attitudes in managing household mask waste.

### Study setting

The research was conducted in DKI Jakarta Province in October-November 2022 by distributing online questionnaires using the Google form platform via social media such as Line, WhatsApp, Instagram, and Twitter.

### Population and sample

This study’s population was public and private university students over 18 years old with Diploma, Bachelor, Master, and Doctoral degrees in the Province of DKI Jakarta, totalling 698,368 students according to the BPS Province of DKI Jakarta in 2022 ( Badan Pusat Statistik Provinsi DKI Jakarta, 2022).

The sample in this study were college students who had filled out a questionnaire according to the inclusion criteria. The samples were collected by purposive sampling using social media such as Line, WhatsApp, Instagram, and Twitter. The number of samples calculated using the Slovin Formula ( Tejada and Punzalan, 2012):

$$n=\frac{N}{1+N\;e^{2}}$$

Notes:

n = required sample size

N = total number of students in DKI Jakarta province

e = selected fault tolerance (0.05)

Based on the results of sample calculations using the above formula, the rounded value of n (number of samples) is 400 samples of college students in DKI Jakarta Province. The inclusion criteria for research subjects were active students enrolled in tertiary institutions with Diploma, Bachelor, Master, and Doctoral degrees in the DKI Jakarta Province who were at least 18 years old and had the ability and willingness to fill out questionnaires distributed via the Google form. Exclusion criteria for research subjects were people who did not have status as students at tertiary institutions in the DKI Jakarta Province and respondents who gave multiple responses, did not fill out the questionnaire completely, and filled out the questionnaire outside the allotted time.

### Data collection

The respondent data was collected using an online questionnaire through the Google Forms platform and distributed using a purposive sampling method via social media, such as Line, WhatsApp, Twitter and Instagram. The target was college students who were studying at universities in DKI Jakarta Province. Of the 967 existing data then cleaning was carried out to determine samples that met the criteria. Then the first 425 respondents who met the inclusion criteria were selected. Respondents who did not meet the desired criteria including missing and incomplete data were removed from the research sample.

### Instruments

The instrument used in this study was an online questionnaire, which respondents filled out themselves using the Google form. A copy of the questionnaire can be found under *Extended data* (Susanna, Dewi, 2023). The questionnaire contained questions on age, gender, education, science groups, place of residence, level of knowledge, attitudes, and behaviours of students in managing household mask waste. The respondent had to approve the informed consent page before the research questionnaire page appeared. The informed consent page stated “All data, both personal data and the results of filling out the questionnaire will be kept confidential and only used for the needs of this research. This research is voluntary and subjects can withdraw at any time and there are no consequences. Completing this questionnaire takes time. about 10-15 minutes, without the risk that can occur during filling out the questionnaire”. The willingness question after the informed consent statement was “Are you willing to fill out this questionnaire?” with the answer options “willing” and “not willing”. If the respondent chose “willing” then they were directed to the questionnaire question page, and if they selected “not willing” they were directed to the last page. After all questions had been answered, the respondent clicked ‘submit’ to submit their response.

The questionnaire was made based on references to the questionnaires in previous research and based on legal aspects, which were then developed and modified by the researchers themselves.

The questionnaire used in this research contained four parts as follows: 1. Respondent’s identity, consisting of name, telephone number, type of e-wallet, age, gender, place of residence, education, name of college, and study program groups; 2. Knowledge about COVID-19; 3. The use of masks; 4. Mask waste management: disinfection of masks, changing the shape of masks, disposal in closed containers, separation of domestic waste, washing hands with soap before and after disposing of masks, and impact of masks waste disposal.

The distributed questionnaire consisted of 66 questions and sociodemographic data, knowledge, attitudes, and behaviours. Respondents who did not meet the desired criteria were removed from the research sample.

### Validity and reliability variables tests

Validity is the accuracy of a measuring instrument in measuring data in a study. This test is needed to determine whether the questions in the questionnaire are irrelevant. Validity and Reliability tests were carried out on 30 respondents. A variable will be said to be valid if the value of the Corrected Item – Total Correlation ≥0.361. The reliability test shows the degree to which the measurement results remain consistent if the measurement is carried out twice or more for the same symptoms and with the same measuring instrument. Reliability can be measured using the Cronbach Alpha method, measured based on a value of 0 to 1. A variable will be reliable if the Cronbach Alpha value shows a result ≥0.6. The results of invalid questions will be deleted so that the final questions total 27 questions on the knowledge variable, 17 on the attitude variable, and 13 on the behaviours variable.

Variables are made binomial in the following way:

Grouping of characteristics respondents are done by making the median the cut-off point because the data distribution is not normal. The groups of characteristics variable are: Age: ≥25 years and <25 years; Gender: Female and Male; Place of residence: Lives alone and Live with family; Education: Postgraduate (master and doctor) and Bachelor & Diploma; Study program groups: Health Clusters (Medicine, Pharmacy, Public Health, Dentistry, Nurse, and other health study programs) and Non-Health Clusters (Science, Social Sciences, Engineering, and Others); Knowledge variables are grouped into two categories: high (if result≥median (22)) and low knowledge (if result<median (22)); Attitude are grouped into two categories: positive (if result≥median (78)) and negative (if result <median (78)); Behaviours are grouped into two categories: Good (if the result≥median (55)) and bad (if the result <median (55)).

### Data analysis

*a. Univariate analysis*

Univariate analysis aims to see the frequency and distribution of each independent and dependent variable. Univariate analysis of the knowledge variable was carried out to see the distribution of the frequency of correct answers to each question to find out how the respondents’ knowledge regarding the management of household mask waste is described. Univariate analysis of the attitude variable was carried out to see the results of the distribution of respondents’ answers to the attitude statement regarding the management of household mask waste. Univariate analysis of behavioural variables was carried out to see the frequency distribution of respondents’ answers to behaviour statements regarding the management of household mask waste.

Univariate analysis was also carried out to see the distribution of the percentage of answers regarding the behaviours of mask waste management in question number 9-13 in the behaviour section for respondents who have high knowledge and live alone. This analysis was conducted to find out which behaviours were most frequently performed by respondents. This distribution analysis was only carried out on the variables of knowledge and place of residence because only these two variables had a relationship with behaviour in this study.

*b. Bivariate analysis*

Bivariate analysis used an association test between the independent and dependent variables. Bivariate analysis was performed using the Chi-Square test because the data used was categorical. Significance if the p-value ≤ α where the significance level α = 0.05, then there is a significant relationship between the independent and dependent variables. Conversely, if p-value > 0.05, there is no significant relationship between variable independents and dependent. Bivariate analysis was performed to determine candidate variables with a p-value ≤ 0.25 to be included in the multivariate analysis. The bivariate analysis also looked at the distribution of respondents’ frequencies and percentages for sociodemographic variables (age, gender, place of residence, education, and scientific background), high knowledge, low knowledge, positive attitudes, negative attitudes, good behaviours, and bad behaviours.

*c. Multivariate analysis*

Multivariate analysis was carried out to see which variables influence student behaviours in managing household mask waste. Multivariate analysis was performed using a logistic regression test with the enter method because it included all predictors into the analysis at once. Variables that will be included in this test are selected by selecting variables with a p-value ≤ 0.25 from the results of the bivariate test. After selecting the candidate variables, the initial modelling of multivariate analysis is carried out. If the results of the significance of the variables tested and the constants in the initial modelling are still > 0.05, then the variable with the greatest significant value is deleted. Logistic regression test was carried out again. If the results of the significance of each variable tested and the results of the significance of the subsequent test constants are ≤0.05 and there is no change in the OR value of more than 10%, then the test results become the final result of multivariate analysis. If the results of the logistic regression test are less than equal to α (p ≤ 0.05) then HI is accepted or there is a relationship between the independent and dependent variables. The exp(β) value determines the magnitude of the influence of a significant independent variable on the dependent variable.

### Ethical considerations

This research has received ethical approval from the Commission of Research and Ethics Faculty of Public Health Universitas Indonesia No. Ket-156/UN2.F10.D11/PPM.00.02.2022. Written informed consent from participants was obtained.

## Results

### a. Frequency distribution of student knowledge variables

An overview of knowledge was generated through univariate tests. Knowledge variables were measured using 18 questions. The following are the results of the descriptive analysis of the respondents’ knowledge (the full dataset can be found under *Underlying data* ( Susanna D, 2023).

Based on Table 1 it is known that most of the respondents have answered correctly. Questions about whether COVID-19 can be infected through droplets from sufferers when they cough or sneeze (Question no. 3) and whether masks that have been wet or dirty must be replaced immediately (Question no. 10) are the questions with the most correct answers (99.1%). The questions with the least number of respondents answered correctly were regarding mask disinfection with the answer choices being heated (31.8%), irradiated (22.4%), and with chemicals, for example chlorine or bleach (35.5%).

**Table 1.** Frequency distribution of respondents based on knowledge variables (n = 425).

Question number	Question	Correct answer	
		Amount	Percentage (%)
1	Severe Acute Respiratory Syndrome Coronavirus-2 (SARS-CoV-2) is the cause of COVID-19	411	96.7
2	COVID-19 can cause respiratory tract disease	418	98.4
3	COVID-19 can be infected through droplets from sufferers when they cough or sneeze	421 *	99.1 *
4	Using a mask properly can prevent transmission of COVID-19	417	98.1
5	How long can the mask be used? (4 hours)	325	76.5
6	Cloth masks should be made of three layers	412	96.9
7	Using a mask must cover the mouth and nose tightly	417	98.1
8	Before using a mask, you must clean your hands with soap and running water or hand sanitizer	412	96.9
9	Used masks cannot be reused	419	98.6
10	Masks that have been wet or dirty must be replaced immediately	421 *	99.1 *
11	Disposable masks should be disposed of immediately after removal	409	96.2
12	Used mask waste should be disposed of separately from domestic waste	410	96.5
13	After throwing away the used mask, you must clean your hands with soap and running water or hand sanitizer	418	98.4
14	What should be done before throwing used disposable masks in the trash?		
14.a	Change the shape	371	87.3
14.b	Discard in a closed trash can	298	70.1
15	How to change the shape of the mask?		
15.a	Cut the masks	379	89.2
15.b	Be destructed	336	79.1
15.c	Ripped off	357	84.0
16	Which are the way to disinfect masks?		
16.a	Heated	135 **	31.8 **
16.b	Irradiated	95 **	22.4 **
16.c	With chemicals, e.g. chlorine or bleach	151 **	35.5 **
17	Which are the right way to wash cloth masks?		
17.a	Using soap or detergent	347	81.6
17.b	Using hot water	234	55.1
17.c	Soaked for 1 minute	242	56.9
18	Which are the impact if used masks are not disposed of properly?		
18.a	Sources of COVID-19 transmission	387	91.1
18.b	Environmental pollution	365	85.9
18.c	Sources of Hazardous Waste	335	78.8

* The highest number of respondents.

** The fewest number of respondents.

### b. Frequency distribution of student attitude variables

An overview of attitudes was generated through a univariate test that was measured using 17 questions selected based on the five statements consisting of strongly disagree, disagree, neutral, agree and strongly agree answers. The following are the results of the analysis of respondents’ statements are shown in Table 2.

**Table 2.** Frequency distribution of respondents based on attitude variables (n = 425).

Statement number	Statement	SD		D		N		A		SA	
		n	%	n	%	n	%	n	%	n	%
1	I believe that COVID-19 is a real disease	3 *	0.7 *	5	1.2	6	1.4	114	26.8	297	69.9
2	Using a mask correctly can reduce the risk of being infected with COVID-19	0	0	2	0.5	30	7.1	104	24.5	289	68.0
3	Using a mask must cover the mouth, nose and chin	0	0	1	0.2	25	5.9	100	23.5	299	70.4
4	Using a medical mask is more effective in reducing the risk of spreading COVID-19 compared to cloth masks	2	0.5	5	1.2	36	8.5	131	30.8	251	59.1
5	Disposable masks should not be reused	0	0	2	0.5	15	3.5	100	23.5	308 *	72.5 *
6	Masks that are dirty or wet should not be reused	3 *	0.7 *	0	0	13	3.1	108	25.4	301	70.8
7	Mask should be changed every 4 hours	1	0.2	6	1.4	31	7.3	150 *	35.3 *	237	55.8
8	Washing cloth masks should use hot water	2	0.5	5	1.2	40	9.4	144	33.9	234	55.1
9	Washing the mask preferably using soap/detergent	1	0.2	7 *	1.6 *	25	5.9	137	32.2	255	60
10	Before the mask is thrown away, it should be cut with scissors	0	0	4	0.9	16	3.8	130	30.6	275	64.7
11	Before the mask is disposed of, it should be disinfected first with disinfectant liquid	0	0	6	1.4	50 *	11.8 *	149	35.1	220	51.8
12	Used masks that are not disposed of properly can be a source of COVID-19 transmission	0	0	4	0.9	25	5.9	126	29.6	270	63.5
13	Used masks that are not disposed of properly can pollute the environment	0	0	1	0.2	18	4.2	114	26.8	292	68.7
14	Used masks that are not deformed by breaking or cutting can increase risk of being reused	0	0	1	0.2	18	4.2	118	27.8	288	67.8
15	Mask waste should be disposed of separately from domestic waste	0	0	3	0.7	26	6.1	143	33.6	253	59.5
16	Masks that have been used must be immediately disposed of in a closed bin	1	0.2	5	1.2	14	3.3	138	32.5	267	62.8
17	After throwing away the masks, you should wash your hands using soap and running water	0	0	2	0.5	19	4.5	119	28	285	67.1

SD = Strongly Disagree, D = Disagree, N = Neutral, A = Agree, SA = Strongly Agree.

* Amount most respondents.

From the 425 respondents who already answered attitude questions, more than 50% of respondents choose the strongly agree option in all questions. Based on the results of the distribution, it can be seen that respondents strongly agree that disposable masks should not be reused (72.5%). There was still 0.7% of respondents who disagreed that COVID-19 is a real disease and masks that are dirty or wet should not be reused.

### c. Frequency distribution of student behaviour variables

An overview of behaviours was generated through a univariate test that was measured using 13 questions selected based on five statements consisting of never, rarely, sometimes, often and always answers. The following are the results of the analysis related to the behaviour of the respondents.

Based on Table 3, respondents always wear masks when going out of the house (70.8%). Respondents still rarely separate mask waste from domestic waste (11.8%). It was found that 16% of respondents had never disinfected masks before they were disposed of.

**Table 3.** Frequency distribution of respondents based on behaviour (n = 425).

Statement number	Statement	N		R		S		O		A	
		n	%	n	%	n	%	n	%	n	%
1	I wear a mask covering mouth, nose and chin	1	0.2	3	0.7	9	2.1	137	32.2	275	64.7
2	I wear a mask when I go out of the house	2	0.5	2	0.5	14	3.3	106	24.9	301 *	70.8 *
3	I do not reuse medical masks	13	3.1	12	2.8	45	10.6	159 *	37.4 *	196	46.1
4	I change the mask if it is already dirty or wet	1	0.2	3	0.7	29	6.8	114	26.8	278	65.4
5	I change the mask every 4 hours	18	4.2	42	9.9	101 *	23.8 *	124	29.2	140	32.9
6	I use hot water for washing cloth masks	46	10.8	40	9.4	80	18.8	110	25.9	149	35.1
7	I use soap/detergent for washing cloth masks	25	5.9	15	3.5	42	9.9	119	28.0	224	52.7
8	I soaked the cloth mask for 1 minute before rinsed	35	8.2	30	7.1	57	13.4	122	28.7	181	42.6
9	I cut or damaged the mask before disposed it	16	3.8	15	3.5	41	9.6	123	28.9	230	54.1
10	I disinfect the mask before disposed it with liquid disinfectant	68 *	16 *	36	8.5	70	16.5	117	27.5	134	31.5
11	I dispose used masks into closed bins	8	1.9	15	3.5	49	11.5	143	33.6	210	49.4
12	I separate masks waste from domestic waste	40	9.4	50 *	11.8 *	64	15.1	125	29.4	146	34.4
13	I wash my hands with soap and running water after disposed my mask	7	1.6	12	2.8	59	13.9	155	36.5	192	45.2

N = Never, R = Rarely (1-9 times a month), S = Sometimes (10-19 times a month), O = Often (20-29 times a month), A = Always (more than 30 times a month).

* The highest number of respondents.

### d. Relationship between knowledge, attitudes, and sociodemographic factors with college students’ behaviour

Bivariate analysis was conducted to see the relationship between the dependent and independent variables. This analysis can also be seen from the distribution of the frequency distribution of respondents from the grouping of knowledge (low and high), attitudes (negative and positive), behaviour (bad and good), and sociodemographic factors (age, gender, place of residence, education, and scientific background). Bivariate analysis was carried out with the Chi-Square test. The results obtained are as follows.

Based on Table 4, the variables that have a significant relationship to behaviours are knowledge and place of residence with p < α (α = 0.05). The resulting p-value for the knowledge variable with behaviour is 0.022 (OR = 1.625, 95%CI = 1.092-2.418) while the place of residence variable has a p-value of 0.008 (OR = 1.722, 95% CI = 1.166-2.542). Attitude variable (p-0.269), age (p-0.557), gender (p-0.762), education (p-0.271), and scientific family (p-0.224) have a p-value > α so that they do not have a significant relationship with behaviour.

**Table 4.** Analysis of the relationship between knowledge, attitudes, and sociodemographic factors with college students behaviour in DKI Jakarta Province.

Variables	Behaviour		p-value	OR (95% CI)
	Bad 210 (49.4%)	Good 215 (50.6%)		
**Knowledge**				
Low	89 (57.1%)	67 (42.9%)	0.022	1.625 (1.092-2.418)
High	121 (45.0%)	148 (55.0%)		
**Attitudes**				
Negative	106 (52.5%)	96 (47.5%)	0.269	1.263 (0.863-1.850)
Positive	104 (46.6%)	119 (53.4%)		
**Age**				
**<**25 years	182 (50.1%)	181 (49.9%)	0.557	1.221 (0.711-2.097)
≥25 years	28 (45.2%)	34 (54.8%)		
**Gender**				
Male	80 (48.2%)	86 (51.8%)	0.762	0.923 (0.625-1.363)
Female	130 (50.2%)	129 (49.8%)		
**Place of residence**				
Living with family	136 (55.1%)	111 (44.9%)	0.008	1.722 (1.166-2.542)
Living alone	74 (41.6%)	104 (58.4%)		
**Education**				
Diploma & Bachelor	182 (50.7%)	177 (49.3%)	0.271	1.395 (0.821-2.371)
Master & Doctor	28 (42.4%)	38 (57.6%)		
**Study Program Group**				
Non-Health Students	140 (47.3%)	156 (52.7%)	0.224	0.756 (0.500-1.145)
Health Students	70 (54.3%)	59 (45.7%)		

OR = Odds Ratio, CI = Confidence interval.

### e. Multivariate analysis results

Multivariate analysis was conducted using multiple logistic regression. The first step is to select variables from bivariate results with a significant level <0.25. The following are the results of the selection of bivariate analysis.

Based on Table 5 shows that there are three variables included in the multivariate candidate: low knowledge, living with family, and non-health students. Bivariate analysis was carried out to select variables that could be used for multivariate analysis, namely variables with a p-value of more than 0.25.

**Table 5.** Multivariate candidates.

Independent variables	p-value	Information
Low knowledge	**0.022** *	Candidate
Negative attitudes	0.269	Not candidate
Age <25 years	0.557	Not candidate
Male	0.762	Not candidate
Living With Family	**0.008** *	Candidate
Diploma & Bachelor	0.271	Not candidate
Non-Health Students	**0.224** *	Candidate

* p-value <0.25 is included in the multivariate candidate.

From the results of the initial multivariate modelling in Table 6, it was found that the non-health students (study program group) variable still had a p-value > 0.05. The next step is to test the confounders by removing the independent variable with the largest p-value.

**Table 6.** Multivariate initial modelling.

Variables	p-value	OR	95% CI
Low knowledge	0.017	1.646	1.093-2.480
Living with family	0.019	1.605	1.080-2.386
Non-health students	0.153	0.731	0.475-1.123
Constant	0.035	0.674	

Based on Table 7 there are no variables with changes in OR > 10%. Thus, the non-health students (study program group) variable is excluded from the modelling analysis. The following are the final modelling results of the logistic regression test:

**Table 7.** The change of OR value without study program group variables.

Variables	p-value	OR Initial	OR New	OR Changed (%)
Low knowledge	0.030	1.646	1.559	5.3
Living with family	0.011	1.605	1.664	3.7
Constant	0.35	0.674	0.625	7.3

Table 8 is the final result of multivariate analysis using multiple logistic regression tests. From the table, the constant values show p-values < 0.05. Knowledge and behaviour variables show a positive regression coefficient with a value of p = 0.030 (OR = 1.559, 95% CI = 1.044-2.330). This result can suggest that students with low knowledge have a tendency to have a bad mask waste management behaviour 1.6 times greater than students who have high knowledge.

**Table 8.** Analysis results of multivariate regression logistics.

Variables	*β*	p-value	OR	95% CI
Low knowledge	0.444	0.030	1.559	1.044-2.330
Living with family	0.509	0.011	1.664	1.124-2.464
Constant	-0.470	0.009	0.625	

Like the knowledge variable, the place of residence variable also shows a positive regression coefficient and significant relationship with behaviour with a p-value=0.011 (OR = 1.664, 95% CI = 1.124-2.464). This result means that students who live with their families are 1.7 times more likely to have bad mask waste management behaviour than those who live alone. The results of logistic regression also show that the OR value of living with family is greater than low knowledge so that the place of residence variable has the greatest influence on student behaviour in managing household mask waste.

## Discussion

### a. Overview of students’ knowledge

Students in this study had high knowledge (63.3%) and more than 90% respondents in 13 questions answered correctly. The easiest questions to answer are number three regarding COVID-19 which can be spread through droplets from sufferers when coughing or sneezing and number 10 regarding masks that have been wet or dirty must be replaced immediately (99.1%). Both of these questions have a high degree of ease for respondents to answer because information about the mode of transmission of COVID-19 and its prevention has been widely disseminated during the pandemic. Transmission of COVID-19 occurs through close contact with an infected person from the respiratory tract when coughing or sneezing ( Zakianis *et al.*, 2021). Preventing transmission through coughing or sneezing can be done by wearing a mask. However, if the mask is dirty or wet and not replaced immediately, it can cause difficulty breathing ( Das *et al.*, 2021). A study on the use of masks found that pathogens on the outer surface of masks that have been wet or dirty have a high risk of causing self-contamination so that masks are no longer effective at protecting against COVID-19 ( Howard *et al.*, 2021).

The most difficult question answered correctly by respondents regarding how to disinfect masks is in question number 16 in options a, b, and c. Only 22.4% chose irradiation, 31.8% heat, and 35.5% used chemicals, such as chlorine or bleach. Various actions including washing, boiling, baking, sun exposure, blowing a hair dryer, autoclaving, alcohol, or ultraviolet irradiation have been recommended to decontaminate mask pathogens. This can be done to protect the environment from pollution due to disposal of masks ( Ma *et al.*, 2020). The Ministry of Health of the Republic of Indonesia has also recommended disinfecting masks before disposal using a disinfectant liquid, chlorine or bleach ( Kementerian Kesehatan RI, 2020). These questions are difficult to answer because information on how to disinfect masks is still difficult to access, both in the community and social media. Because of this, students need to increase their knowledge, especially in the management of mask waste because they are considered one of the main forces for raising awareness among families and communities.

The knowledge gap can have an adverse effects during a pandemic because it can increase confusion in society ( Noreen *et al.*, 2020). University students are widely described as opinion leaders in their local communities when it comes to health because of their perceived high levels of health literacy ( Baker *et al.*, 2021). The students in this study showed that they have a high level of literacy as well as technological advances in today’s information dissemination, especially from social media, which enabled them to answer almost every question correctly.

### b. Overview of students’ attitudes

Students’ attitudes show positive results (52.5%). Most of them have a positive attitude towards using a mask that must cover their mouth, nose and chin (70.4%), agree that disposable masks cannot be reused (72.5%), and masks that are dirty or wet should not be reused (70.8%). These results indicate that respondents have a high awareness of using a good mask. The World Health Organization has recommended using a mask by covering the nose, mouth and chin and not reusing disposable masks. When the mask is dirty and wet it must be replaced immediately ( World Health Organization, 2020b).

Masks that must be replaced every four hours in question number seven became the statement with the most agree answer choices (35.3%). Masks can only effectively be used within 3-4 hours so if it has exceeded that time it should be replaced ( Prata *et al.*, 2021). The Ministry of Health of the Republic of Indonesia also recommends wearing masks for no more than four hours and always providing spare masks ( Kementerian Kesehatan RI, 2021). In the statement regarding masks that must be disinfected with disinfectant liquid before being disposed of, it was found that 11.8% of respondents chose neutral and this was the highest percentage in the neutral choice. This shows that there are still 11.8% of respondents who do not realize the importance of disinfecting masks before disposal. Disinfection is necessary to reduce decontamination in the environment ( Ma *et al.*, 2020).

Attitudes toward washing masks with soap/detergent still found 1.6% of respondents choosing to disagree. The World Health Organization has recommended washing masks using soap/detergent. This has to be done to reduce virus contamination found in masks ( World Health Organization, 2020b). The Indonesian Ministry of Health through the Directorate of Health Promotion and Community Empowerment has distributed flyers on how to clean cloth masks, such as using soap and detergent ( Direktorat Promosi Kesehatan Dan Pemberdayaan Masyarakat Kementerian Kesehatan RI, 2020). Increasing information and counselling from the government regarding mask disinfection and how to wash masks needs to be done so that a more positive attitude can be built and evenly shared by every student.

The question on whether COVID-19 is a real disease in question number one was the statement with the most strongly disagree answers (0.7%). Public distrust in the presence of COVID-19 can be caused by false information that is spread on various platforms. In the midst of a situation that makes people restless and sad, fake news about the coronavirus was circulating in various media, especially social media. The Ministry of Communication and Information on 17 April 2022 recorded 5,829 hoaxes about COVID-19 circulating in the media ( Kementerian Komunikasi dan Informatika, 2022). In addition to these statements, the statement about masks that are dirty or wet should not be reused is also the statement with the most strongly disagree answers (0.7%) even though 70.8% of respondents voted strongly agree. When masks get wet or dirty, they should be changed immediately and should not be worn for a long time ( Das *et al.*, 2021). The need for masks, which then becomes higher when the replacement of masks is more frequent, allows for reluctance to purchase masks, especially among students.

### c. Overview of students’ behaviour

The majority of students already have good behaviour (50.6%), but the percentage of students with bad behaviours is not too different. Thus, it can be said that there are still many students who have bad behaviour. The majority of respondents indicated that they always wear masks when going out of the house (70.8%). Since the COVID-19 pandemic was announced by WHO, everyone has been advised to wear masks, especially when going out of the house ( World Health Organization, 2020a). In question number three regarding not reusing medical masks, the percentage of answer choices often is the most chosen by respondents (37.4%). There are still 23.8% of students sometimes changing masks every four hours. Not reusing medical masks and changing masks every four hours has been recommended by WHO because reusing medical masks is ineffective and allows viruses and bacteria to remain ( World Health Organization, 2020a).

The behaviour of separating mask waste from domestic waste was found by 11.8% respondents who rarely practiced it. Mask waste is included in infectious waste which must go through special treatment and a sorting process separately from other waste before being disposed of in the domestic waste bin ( Sumiarsih and Rasniah Sarumi, 2021). The Ministry of Environment and Forestry of the Republic of Indonesia has issued a circular to the public which advises to separate mask waste from domestic waste ( Menteri Lingkungan Hidup dan Kehutanan Republik Indonesia, 2021). Facilities and infrastructure for sorting mask waste from domestic waste are inadequate so it is still difficult to carry out with students in this study. Solutions are needed to managed mask waste so it does not end up in the environment mixed with other domestic waste and become a source of hazardous waste.

There were also respondents who had poor behaviour in disinfecting masks before disposal. As many as 16% of students have never disinfected masks before disposing them. Mask disinfection before disposal is necessary to reduce contamination in the environment ( Rubio-Romero *et al.*, 2020). However, students’ knowledge, attitudes and behaviour towards mask disinfection is still poor. There needs to be education around the easiest methods for the community to disinfect masks, such as by using bleach, floor cleaning liquid, or disinfectant liquid which are widely available.

### d. Relationship between knowledge, attitudes, and sociodemographic factors with students’ behaviour

Knowledge shows a significant relationship with students’ behaviour and is positively correlated with p=0.030 (OR = 1.559, 95% CI = 1.044-2.330). The students with low knowledge have a 1.6 times greater risk of poor behaviour in managing mask waste compared to those with high knowledge. This research is in line with research conducted in Vietnam that found there is a relationship between knowledge and behaviour with a p value <0.001 ( Le An *et al.*, 2021).

The group of respondents with good knowledge was also seen to frequently engage in mask waste management behaviour. From the results of the distribution, it was found that 57.6% of students with good knowledge had often damaged masks by cutting or tearing them, 32.7% had often disinfected masks before disposal, 52.8% had often disposed of mask waste in a closed bin, 34.2% had often separated mask waste with domestic waste, and 47.6% have washed their hands with soap and running water after disposing of masks.

This finding illustrates that the majority of respondents with high knowledge have often performed good behaviour in managing household mask waste. This explains that people who are highly knowledgeable tend to have better behaviour. Therefore, it is necessary to increase student knowledge about better management of mask waste to encourage behaviour. Counselling and education in increasing knowledge can be carried out through various media such as print media, electronic media, social media, to peer group approaches, and instructions or appeals from Regional Heads, both Governors and Regents/Mayors ( Retnaningsih *et al.*, 2020).

In this study, no significant relationship was found between attitudes and behaviour, so the hypothesis was rejected. This suggests that the level of behaviour may not increase even though their attitude is better. For example, the majority of respondents strongly agree that it is their responsibility to disinfect first with a disinfectant liquid before disposing of the mask (51.8%) but they might not do it properly. This may also occur due to a misunderstanding of the respondent in answering so that the results shown are not the actual attitude of the student. This result is inconsistent with the results of previous research which found a significant correlation between attitudes and behaviour, which explains that those with better attitudes will also have better behaviour ( Islam *et al.*, 2021).

The results of the analysis shows that the p-value of the age variable is 0.557 so that there is no significant relationship between age and behaviour. This is also seen in the variables of gender, education, and scientific family which produce a p-value>0.05 so that they do not have correlation with behaviour. The previous research conducted by Sulistyawati (2021) showed that there was a relationship between gender and behaviour but there was no relationship between age and education ( Sulistyawati *et al.*, 2021). Another study conducted by Le An *et al.* (2021) resulted in a relationship between gender and behaviour but there was no significant relationship between age, education level, and major or scientific groups (An *et al.*, 2021). Health students should be the one who have better mask waste management behaviour. However, in this study, no relationship was found between scientific group and behaviour. This might happen because knowledge about managing mask waste in households is also still low. Information and socialization of policies regarding the management of mask waste is still very rare. Facilities and infrastructure in Indonesia are also inadequate so that students from both the health group and non-health group do not have a different influence on behaviour.

Of the five sociodemographic factors, only the residence variable has a significant and positive correlation with students’ behaviour in managing mask waste. The analysis resulted in a p-value of 0.011 with an OR of 1.664 (1.124-2.464). The hypothesis in this case is accepted. Students who live with their families have a 1.7 times greater tendency to behave badly compared to students who live alone. The residence variable is the variable with the greatest influence on the behaviour of mask waste management followed by the knowledge variable. This result is not in line with research in Vietnam which did not have a significant relationship between place of residence and behaviour (p = 0.653) ( Le An *et al.*, 2021). Research in Korea also shows no relationship between where you live and behaviour ( Lee, Kang and You, 2021).

Respondents who live alone already have good mask waste management behaviour. From the results of the distribution analysis in a group of students who live alone and behaviour, it was found that 51.7% of students had always destroyed masks before they were disposed of, 35.4% had always disinfected, 48.9% had always disposed of them in closed bins, 38.2% had always separated them from domestic waste, and 38.8% had washed their hands frequently after disposing of the mask. The influence of the family when students live together affects their behaviour because they tend not to have their own autonomy and depend on the rules made by their parents ( Hatabu *et al.*, 2020). Living alone is one of the considerations for students who can show stronger self-control and take safer actions than others. Research conducted in Japan shows that place of residence is a significant factor in demonstrating safer behaviour ( Hatabu *et al.*, 2020). The place of residence variable is also the variable that has the greatest influence on the behaviour of household mask waste management.

Increasing knowledge is needed as a way to make people aware of the importance of managing household mask waste and encourage better behaviour. In the current situation, the government is experiencing difficulties in dealing with medical waste that appears from both hospitals and households. The public should be aware that if disposed of improperly, this waste can still carry traces of viral contaminants and can cause serious problems in the future ( Dharmaraj *et al.*, 2021). Policies related to the management of mask waste need to be tightened and balanced with adequate infrastructure so that behaviour improvement in the community can be carried out properly. Education on infectious waste management is also needed to increase public knowledge, awareness, and ability to sort, minimize, destroy, collect, package, and store waste properly ( Sari *et al.*, 2021). This is because mask waste that ends up in the environment can carry harmful pathogens that can pollute the environment ( Du, Huang and Wang, 2022). In the case of the COVID-19 pandemic, this virus can survive for three days on plastic surfaces so that it can become a source of transmission for COVID-19 ( Salian *et al.*, 2021).

The strength in this study lies in the target population which was college students who are widely seen as a generation with high health literacy among their friends and social environment. There is also little research on the management of mask waste in Indonesia so this study can become a reference in making policies and more scientific approaches can be adopted to facilitate mask waste control. The participation of the central and regional governments is very important in monitoring and coaching the community. Without policies and government participation in terms of the importance of managing mask waste before it is disposed of in the community, it will be difficult to create good habits that can be carried out by the community in the household.

This study still has limitations because data collection was carried out online so that there is a potential for result bias and errors in interpretation of the questionnaire questions. To overcome this, questions in the questionnaire gave information and use language that was easier to understand. Validity and reliability tests were carried out to ensure that the data obtained was of high quality. The sample size may be sufficient for statistical analysis but the results can be more representative if a larger sample is used so further research is needed to solve this problem. Further research can also be carried out by analysing other factors that can influence the behaviour of mask waste management.

## Conclusion

College students in DKI Jakarta Province tend to have high knowledge, positive attitudes, and good behaviour in managing household mask waste. Knowledge and place of residence have a significant relationship with behaviour. However, attitude, age, gender, education and scientific group did not show a significant relationship with behaviour. The higher the student’s knowledge, the better behaviour will be encouraged. Students who live alone show better behaviour compared to students who live with their families. The place of residence variable has the greatest influence on the behaviour of mask waste management at the household, followed by the knowledge variable.

Following the findings of this research, some recommendations were addressed for the governance of DKI Jakarta Province. The Provincial Government of DKI Jakarta can conduct outreach and education to the public both directly and digitally to provide information on how to properly manage household mask waste and improve facilities and infrastructure in the community environment so that mask waste management can be carried out properly in accordance with regulation. Policies that can support the improvement of mask waste management in households is also needed. Further research can examine variables and other factors related to the behaviours around household mask waste management and develop educational media regarding the management of mask waste.

## Data Availability

Dryad: Relationship of knowledge, attitudes, and sociodemographic factors with behavior in the management of mask waste in households. https://doi.org/10.5061/dryad.8931zcrvm ( Susanna D, 2023). This project contains the following underlying data:
-
DATA_425_RECODE.sav-_NEW1.sav This project contains the following extended data:
- Questionnaire.docx Data are available under the terms of the Creative Commons Zero “No rights reserved” data waiver (CC0 1.0 Public domain dedication).
