# Supplementary material for: Knowledge, attitudes, and behaviour of college students in disposing used masks during the COVID-19 pandemic in DKI Jakarta Province
Source: F1000Res. 2024 Mar 4;12:511. Originally published 2023 May 17. [Version 3] doi: 10.12688/f1000research.130691.3 (PMC10918313; doi:10.12688/f1000research.130691.3)
Supplement: Peer review history

# Reviewer report: Reviewer response for version 3

**DOI:** 10.5256/f1000research.163212.r252494

**Published:** 2024-03-06

**Authors:** Armin Šljivo

**Affiliations:** 1 University of Sarajevo, Sarajevo, Federation of Bosnia and Herzegovina, Bosnia and Herzegovina

Dear Author,

 Thank You very much for the extensive revision. The paper now is clarified in all terms and presents the research in an exemplary manner. Your meticulous attention to detail and insightful suggestions have significantly enhanced the overall quality of the paper. I am sincerely grateful for the time and effort you dedicated to revising the manuscript.

 Your constructive feedback has not only clarified various aspects of the research but has also strengthened the coherence of the paper. The thoroughness with which you approached the revision process reflects your expertise and commitment to ensuring the highest standards of scholarly work.

 I would also like to express my gratitude for the promptness with which you completed the revision. Your efficiency has been instrumental in advancing the progress of this research, and I am truly appreciative of your timely and valuable contributions.

 Once again, thank you for your dedication to refining this work. I am confident that your valuable input will contribute significantly to the success of this research endeavor. I look forward to any further insights you may have during subsequent stages of the publication process.

Is the work clearly and accurately presented and does it cite the current literature?

Partly

If applicable, is the statistical analysis and its interpretation appropriate?

Yes

Are all the source data underlying the results available to ensure full reproducibility?

Partly

Is the study design appropriate and is the work technically sound?

Yes

Are the conclusions drawn adequately supported by the results?

Partly

Are sufficient details of methods and analysis provided to allow replication by others?

Partly

Reviewer Expertise:

Public Health and COVID-19

I confirm that I have read this submission and believe that I have an appropriate level of expertise to confirm that it is of an acceptable scientific standard.

# Reviewer report: Reviewer response for version 2

**DOI:** 10.5256/f1000research.160369.r238728

**Published:** 2024-02-16

**Authors:** Armin Šljivo

**Affiliations:** 1 University of Sarajevo, Sarajevo, Federation of Bosnia and Herzegovina, Bosnia and Herzegovina

Dear Authors,

 I express my gratitude for your thorough revision of the paper titled "Knowledge, attitudes, and behavior of college students in disposing used masks during the COVID-19 pandemic in DKI Jakarta Province." You have successfully addressed all the proposed changes outlined in Revision 1. The paper is now significantly clearer and more readable.

 Nevertheless, there remains an uncertainty regarding how the prevention of multiple responses was managed on the Google Forms platform. If the option of restricting responses to one per email or any other software preventing multiple responses per IP address was not employed, I recommend providing clarification on this matter in the Limitations section. In future studies, I recommend utilizing software that ensures the prevention of multiple responses per IP address or email. Failing to implement such measures could potentially introduce bias or confounding factors, as authors may submit multiple responses.

 Also, in the Limitations section please explain or rephrase the sentence "This research is using online platform, so it is easier to reach many respondents and cheaper" because it doesn't pose a Limitations, rather a Strength of the study.

 Thank you once again for your dedication to improving the quality of the manuscript.

 Sincerely

Is the work clearly and accurately presented and does it cite the current literature?

Partly

If applicable, is the statistical analysis and its interpretation appropriate?

Yes

Are all the source data underlying the results available to ensure full reproducibility?

Partly

Is the study design appropriate and is the work technically sound?

Yes

Are the conclusions drawn adequately supported by the results?

Partly

Are sufficient details of methods and analysis provided to allow replication by others?

Partly

Reviewer Expertise:

Public Health and COVID-19

I confirm that I have read this submission and believe that I have an appropriate level of expertise to confirm that it is of an acceptable scientific standard, however I have significant reservations, as outlined above.

# Reviewer report: Reviewer response for version 2

**DOI:** 10.5256/f1000research.160369.r238727

**Published:** 2024-02-16

**Authors:** Narisara Kaewchutima, Nopadol Precha

**Affiliations:** 1 Department of Environmental Health and Technology, Walailak University, Thai Buri, Nakhon Si Thammarat, Thailand; 2 Environmental health, Walailak University, Thai Buri, Nakhon Si Thammarat, Thailand

Dear Authors,

 Thank you very much for your response. I have no further comments, all tasks were addressed.

Is the work clearly and accurately presented and does it cite the current literature?

Partly

If applicable, is the statistical analysis and its interpretation appropriate?

I cannot comment. A qualified statistician is required.

Are all the source data underlying the results available to ensure full reproducibility?

Yes

Is the study design appropriate and is the work technically sound?

Yes

Are the conclusions drawn adequately supported by the results?

Partly

Are sufficient details of methods and analysis provided to allow replication by others?

Yes

Reviewer Expertise:

Waste management/ Environmental management/ Environmental policy

We confirm that we have read this submission and believe that we have an appropriate level of expertise to confirm that it is of an acceptable scientific standard.

# Reviewer report: Reviewer response for version 1

**DOI:** 10.5256/f1000research.143464.r214918

**Published:** 2023-10-18

**Authors:** Narisara Kaewchutima, Nopadol Precha

**Affiliations:** 1 Department of Environmental Health and Technology, Walailak University, Thai Buri, Nakhon Si Thammarat, Thailand; 2 Environmental health, Walailak University, Thai Buri, Nakhon Si Thammarat, Thailand

Dear authors,

 After reviewing through the article, it is interesting study particularly in the used facemask management in university students. However, I have some comments for giving more discussion in detail as following:

 - Discussion:

 Although, several studies have recommended cutting facemasks into small pieces before discarding them to avoid reuse. However, the used facemasks are infectious waste, cutting them into small pieces may increase the volumes of infectious waste in the environment. So, this practice may also complicate waste disposal and have many consequences, particularly for humans and the environment. You may discuss more in this issue, because it is one of an important practices in facemask management.

 - Discussion:

 To further clarify the impact of a student's place of residence on facemask disposal behavior, it would be beneficial to provide additional details regarding the observed difference in behavior between students who live with their families and those who live alone. Specifically, it is essential to elaborate on the definition of "living alone." Does this pertain to students who reside in university dormitories or those who have separate accommodations in houses or apartments? The distinction is crucial because, in university dormitories or private housing arrangements, facilities such as specific disposal bins may be available. The provision of these amenities could encourage students to consistently dispose of facemasks in the designated bins, ultimately promoting better disposal practices. These could be beneficial to support your result.

 - Discussion:

 If considered relevant, the authors could present more perspective of knowledge and practice among university students during the pandemic from the more or less same demographic (in this case, e.g., Southeast Asia). These following papers could be considered to be cited: https://doi.org/10.1371/journal.pone.0284492

 Thank you

Is the work clearly and accurately presented and does it cite the current literature?

Partly

If applicable, is the statistical analysis and its interpretation appropriate?

I cannot comment. A qualified statistician is required.

Are all the source data underlying the results available to ensure full reproducibility?

Yes

Is the study design appropriate and is the work technically sound?

Yes

Are the conclusions drawn adequately supported by the results?

Partly

Are sufficient details of methods and analysis provided to allow replication by others?

Yes

Reviewer Expertise:

Waste management/ Environmental management/ Environmental policy

We confirm that we have read this submission and believe that we have an appropriate level of expertise to confirm that it is of an acceptable scientific standard, however we have significant reservations, as outlined above.

## References

[1] : Knowledge and practice of facemask disposal among university students in Thailand: A new normal post the COVID-19 pandemic. *PLoS One* .2023;18(4) : 10.1371/journal.pone.0284492 e0284492 10.1371/journal.pone.0284492 37053157 PMC10101522

# Response

**Published:** 2023-12-13

**Authors:** Dewi Susanna

**Affiliations:** Universitas Indonesia, Indonesia

**Responses to the Comments’ Reviewer-1**

 Dear

**Narisara Kaewchutima**

 Department of Environmental Health and Technology, Walailak University, Thai Buri, Nakhon Si

 Thammarat, Thailand

**Nopadol Precha**

 Environmental Health, Walailak University, Thai Buri, Nakhon Si Thammarat, Thailand

 Thank you very much for assessing our manuscript.

 Here are our responses in addressing point by point of your comments.

***The comments and responses:***

 Dear authors,

 After reviewing through the article, it is interesting study particularly in the used facemask management in university students. However, I have some comments for giving more discussion in detail as following:

 - Discussion:

 Although, several studies have recommended cutting facemasks into small pieces before discarding them to avoid reuse. However, the used facemasks are infectious waste, cutting them into small pieces may increase the volumes of infectious waste in the environment. So, this practice may also complicate waste disposal and have many consequences, particularly for humans and the environment. You may discuss more in this issue, because it is one of an important practices in facemask management.

Response:

 Cutting facemasks after using it is one of the ways to prevent from reuse. This way may increase the volumes of infectious waste in the environment so that is why the government's role in effective management of increasing medical waste is needed. We already add more information in page 16 (word document) as additional information.

*A constructive methodology or system must be adopted so that the increasing management of medical waste can be carried out effectively in every country in the world. Adequate waste recycling or its conversion to other energy forms may be an excellent solution that can be implemented by any government to control the current situation. Common techniques used for the COVID-19 waste treatment include the Incineration, Physical and Chemical methods. These techniques are applied to handle the different types of waste that are carried by the COVID-19.* *An alternative way to overcome the problem is improvised face mask production either made through nanotechnology or biomaterial instead of microplastic and should be completely biodegradable so that it may not harm the terrestrial as well as the marine ecosystem (Dharmaraj et al., 2021b).*

 - Discussion:

 To further clarify the impact of a student's place of residence on facemask disposal behavior, it would be beneficial to provide additional details regarding the observed difference in behavior between students who live with their families and those who live alone. Specifically, it is essential to elaborate on the definition of "living alone." Does this pertain to students who reside in university dormitories or those who have separate accommodations in houses or apartments? The distinction is crucial because, in university dormitories or private housing arrangements, facilities such as specific disposal bins may be available. The provision of these amenities could encourage students to consistently dispose of facemasks in the designated bins, ultimately promoting better disposal practices. These could be beneficial to support your result.

Response:

 The additional explanation on page 16.

*In this study, living alone define students that are not live with their family whether they live in dormitory, in house, or apartments.*

 - Discussion:

 If considered relevant, the authors could present more perspective of knowledge and practice among university students during the pandemic from the more or less same demographic (in this case, e.g., Southeast Asia). These following papers could be considered to be cited: https://doi.org/10.1371/journal.pone.0284492

Response:

 Thank you for your reference, we’ve added more information from those study case to introduction page 2 and 19 (word document)

*Research conducted on students in Thailand shows that there was a positive correlation between facemask disposal knowledge and practices (p < 0.01) (Kaewchutima et al., 2023) A study conducted in 2021, shows a statistically significant association found among knowledge level and educational qualification (p < 0.0001) and gender (p < 0.001) (Jalal et al., 2021).*

*Jalal, S.M. et al. (2021) ‘Assessment of knowledge, practice and attitude about biomedical waste management among healthcare professionals during COVID-19 crises in Al-Ahsa’, Healthcare (Switzerland), 9(6). Available at: https://doi.org/10.3390/healthcare9060747.*

*Kaewchutima, N. et al. (2023) ‘Knowledge and practice of facemask disposal among university students in Thailand: A new normal post the COVID-19 pandemic’, PLoS ONE, 18(4 APRIL). Available at: https://doi.org/10.1371/journal.pone.0284492.*

 Thank you for your valuable comments and suggestions.

 Best regards,

 Authors.

# Reviewer report: Reviewer response for version 1

**DOI:** 10.5256/f1000research.143464.r208840

**Published:** 2023-10-06

**Authors:** Armin Šljivo

**Affiliations:** 1 University of Sarajevo, Sarajevo, Federation of Bosnia and Herzegovina, Bosnia and Herzegovina

The paper entitled "Knowledge, attitudes, and behaviour of college students in disposing used masks during the COVID-19 pandemic in DKI Jakarta Province"  provides us information regarding the other side of the COVID-19 pandemic. The paper is robust and need further clarification on some points.

 Comments:

**Abstract.**

 Firstly, I would advise the authors to use more complex sentence structures, as the first two sentences of the Background part could be merged.

 Secondly, in the Results section please put p= instead of p-value=, as we all know what p stands for.

 Thirdly, put keywords according to the MESH browser.

**Introduction: **Just see if there are other similar research papers like this one, and add data from it.

**Methods.**

 Firstly, does this study have an Ethical approval and if so, please add it in the Manuscript. If not, explain why and why doesn't it need the Ethical approval.

 Secondly, add us the explanation how did You manage to prevent multiple responses from one individual, as the platforms used in this setting doesn't prevent multiple responses. And if You used the one answer per e-mail method, please provide us the explanation how was anonymity preserved.

 Thirdly, I would advise You to give us the example of the study questioner either in Table or Supplement and to give us information regarding the internal consistency. You have explained it in the Methods, but the numbers should be added.

 Overall, this part needs and extensive clarification also in the Limitations part of the Discussion as some points have flaws which could further pose a bias or a confounding. 

**Results: **They are appropriate

**Discussion:** Please add more literature and references and the futures of this study.

Is the work clearly and accurately presented and does it cite the current literature?

Partly

If applicable, is the statistical analysis and its interpretation appropriate?

Yes

Are all the source data underlying the results available to ensure full reproducibility?

Partly

Is the study design appropriate and is the work technically sound?

Yes

Are the conclusions drawn adequately supported by the results?

Partly

Are sufficient details of methods and analysis provided to allow replication by others?

Partly

Reviewer Expertise:

Public Health and COVID-19

I confirm that I have read this submission and believe that I have an appropriate level of expertise to confirm that it is of an acceptable scientific standard, however I have significant reservations, as outlined above.

# Response

**Published:** 2023-12-13

**Authors:** Dewi Susanna

**Affiliations:** Universitas Indonesia, Indonesia

​​​​​​ *Dear **Armin Šljivo,***

 Thank you very much for your comments and recommendations for improvement.

 Here are we respond point by point as mentioned below:

*Comments:*

**1. Abstract.**

 a. Firstly, I would advise the authors to use more complex sentence structures, as the first two sentences of the Background part could be merged. 

Responses:

 We have paraphrased it with a more complex sentence in the background section of the abstract (page 1).

*The COVID-19 pandemic has increased the need for mask production which has caused the problem of mask waste generating in the environment without being managed.*

 b. Secondly, in the Results section please put p= instead of p-value=, as we all know what p stands for.

Responses:

 We have revised some parts to use “p=” instead of “p-value=” (page 1, on result section of the abstract)

*Statistically, there was a significant relationship between knowledge and behaviours (p = 0.022), but there was no significant relationship between attitudes and behaviours (p = 0.269). In addition, the sociodemographic factor variables showed a significant relationship between place of residence and behaviours (p = 0.008). However, there was no significant relationship between age, gender, education, and study program groups and behaviours (p > 0.05).*

*Also, in the text, all the changes of p value to be marked in yellow block.*

 c.  Thirdly, put keywords according to the MESH browser.

Responses:

 We have revised the keywords using MESH.

*COVID-19, knowledge, attitudes, practice, sociodemographic factors, students, waste management, cross-sectional studies.*

**2.  Introduction:**

  Just see if there are other similar research papers like this one, and add data from it. 

Responses:

 We have added similar research papers on introduction and added data from it.

*Research conducted on students in Thailand shows that there was a positive correlation between facemask disposal knowledge and practices (p < 0.01) (Kaewchutima et al., 2023) A study conducted in 2021, shows a statistically significant association found among knowledge level and educational qualification (p < 0.0001) and gender (p < 0.001) (Jalal et al., 2021).*

**3. Methods.**

 a. Firstly, does this study have an Ethical approval and if so, please add it in the Manuscript. If not, explain why and why doesn't it need the Ethical approval. 

Responses:

 We have added the ethical approval number on page 17 (word document)

*This research has received ethical approval from the Commission of Research and Ethics Faculty of Public Health Universitas Indonesia No. Ket-156/UN2.F10.D11/PPM.00.02.2022.*

 b. Secondly, add us the explanation how did You manage to prevent multiple responses from one individual, as the platforms used in this setting doesn't prevent multiple responses. And if You used the one answer per e-mail method, please provide us the explanation how was anonymity preserved.  

Responses:

*We carried out screening by selecting samples that fit the inclusion criteria including sorting based on their email. Apart from that, we carry out cleaning by using a frequency distribution list. We only keep this data as a reference to differentiate samples, but we do not process their personal data so that we can ensure that each respondent's data will not be spread out*

 c.  Thirdly, I would advise You to give us the example of the study questioner either in Table or Supplement and to give us information regarding the internal consistency. You have explained it in the Methods, but the numbers should be added.

Responses:

 We’ve added questionnaire on appendix 1 as additional information in this sheet.

 Overall, this part needs and extensive clarification also in the Limitations part of the Discussion as some points have flaws which could further pose a bias or a confounding.

Responses:

 We’ve added strength and limitation on page 17 (word document).

*This research is using online platform, so it is easier to reach many respondents and cheaper. This research is also using a cross-sectional study so that it is able to explain the relationship between one variable and other variables in the population. However, it does not have the ability to explain the dynamics of changes in conditions in different time periods.*

 Thank you for your valuable comments and suggestions.

 Best regards,

 Authors.

 Appendix 1: Questionnaire

****

**First Part - The Informed Consent**

 “All data, both personal data and the results of filling out the questionnaire will be kept confidential and only used for the needs of this research. This research is voluntary and subjects can withdraw at any time and there are no consequences. Filling in this questionnaire takes time. about 10-15 minutes, without the risk that can occur during filling out the questionnaire".

 The willingness question after the informed consent statement was.

 "Are you willing to fill out this questionnaire?" with the answer options "willing" and "not willing". If the respondent chooses "willing" then they will be directed to the questionnaire question page, while if the answer is "not willing" they will be directed to the last page.

**Second Part – Personal Information**

 Respondent’s Name

 Gender (male, female)

 Age (18-24 y.o, 25-32 y.o, >32 y.o)

 Domicile (Jabodetabek, Outside Jabodetabek)

 Place of Residence

 Are you a student?

**Third Part – Respondent’s Education Information**

 Education Level (Diploma, S1, S2, S3)

 University Location (Jakarta, Depok, Bekasi, Tangerang, etc)

 Name of college

 Study program groups

**Fourth Part - Knowledge Regarding COVID-19 and Mask Waste Management (True or False)**

1. Severe Acute Respiratory Syndrome Coronavirus-2 (SARS-CoV-2) is the cause of COVID-19
2. COVID-19 can cause respiratory tract disease
3. COVID-19 can be infected through droplets from sufferers when they cough or sneeze
4. Using a mask properly can prevent transmission of COVID-19
5. How long can the mask be used? (4 hours)
6. Cloth masks should be made of three layers
7. Using a mask must cover the mouth and nose tightly
8. Before using a mask, you must clean your hands with soap and running water or hand sanitizer
9. Used masks cannot be reused
10. Masks that have been wet or dirty must be replaced immediately
11. Disposable masks should be disposed of immediately after removal
12. Used mask waste should be disposed of separately from domestic waste
13. After throwing away the used mask, you must clean your hands with soap and running water or hand sanitizer
14. What should be done before throwing used disposable masks in the trash?
  1. Change the shape
  2. Discard in a closed trash can
15. How to change the shape of the mask?
  1. Cut the masks
  2. Be destructed
  3. Ripped off
16. Which are the way to disinfect masks?
  1. Heated
  2. Irradiated
  3. With chemicals, e.g. chlorine or bleach
17. Which are the right way to wash cloth masks?
  1. Using soap or detergent
  2. Using hot water
  3. Soaked for 1 minute
18. Which are the impact if used masks are not disposed of properly?
  1. Sources of COVID-19 transmission
  2. Environmental pollution
  3. Sources of Hazardous Waste

**Fifth Part – Attitudes Regarding COVID-19 and Mask Waste Management**

 Notes: five statements consisting of strongly disagree, disagree, neutral, agree and strongly agree answers

1. I believe that COVID-19 is a real disease
2. Using a mask correctly can reduce the risk of being infected with COVID-19
3. Using a mask must cover the mouth, nose and chin
4. Using a medical mask is more effective in reducing the risk of spreading COVID-19 compared to cloth masks
5. Disposable masks should not be reused
6. Masks that are dirty or wet should not be reused
7. Mask should be changed every 4 hours
8. Washing cloth masks should use hot water
9. Washing the mask preferably using soap/detergent
10. Before the mask is thrown away, it should be cut with scissors
11. Before the mask is disposed of, it should be disinfected first with disinfectant liquid
12. Used masks that are not disposed of properly can be a source of COVID-19 transmission
13. Used masks that are not disposed of properly can pollute the environment
14. Used masks that are not deformed by breaking or cutting can increase risk of being reused
15. Mask waste should be disposed of separately from domestic waste
16. Masks that have been used must be immediately disposed of in a closed bin
17. After throwing away the masks, you should wash your hands using soap and running water

**Sixth Part – Behaviour Regarding COVID-19 and Mask Waste Management**

 Notes: five statements consisting of never, rarely, sometimes, often and always answers

1. I wear a mask covering mouth, nose and chin
2. I wear a mask when I go out of the house
3. I do not reuse medical masks
4. I change the mask if it is already dirty or wet
5. I change the mask every 4 hours
6. I use hot water for washing cloth masks
7. I use soap/ detergent for washing cloth masks
8. I soaked the cloth mask for 1 minute before rinsed
9. I cut or damaged the mask before disposed it
10. I disinfect the mask before disposed it with liquid disinfectant
11. I dispose used masks into closed bins
12. I separate masks waste from domestic waste
13. I wash my hands with soap and running water after disposed my mask

Thank you very much again for reviewing this manuscript for improvement.

 Best regards,

 Authors.
